# A Full Uterus: Hematometra from Cervical Scarring

**DOI:** 10.5811/cpcem.2019.10.44925

**Published:** 2020-01-24

**Authors:** Christopher S. Sampson, Krysta Arnold

**Affiliations:** University of Missouri School of Medicine, Department of Emergency Medicine, Columbia, Missouri

## Abstract

A 29-year-old female presented with abdominal pain, nausea, and vomiting. She reported no menstrual period for one year. She did report monthly episodes of severe cramping. A loop electrosurgical excision procedure was performed approximately 10 months prior. On pelvic exam, a smooth cervix with scarring over the os was visualized with no evidence of cervical opening. A pelvic ultrasound showed an enlarged uterus with contents within the endometrial cavity likely representing hemorrhage of different ages and ongoing bleeding. Gynecology was consulted and performed an incisional opening of the cervix. The patient was diagnosed with hematometra from scarred cervical os.

## CASE PRESENTATION

A 29-year-old female presented with abdominal pain, nausea, and vomiting for one day. The pain was in the lower abdomen and described as an achy, constant, fluctuating pain. She had a negative home pregnancy test one week prior and reported no menstrual period for one year due to her current breast feeding. She did report monthly episodes of severe cramping. Vital signs were within normal limits. On exam she was noted to have a soft, non-distended abdomen. Mild, suprapubic tenderness was appreciated. On pelvic exam, a smooth cervix with scarring over the os was visualized with no evidence of cervical opening.

**Image 1:**
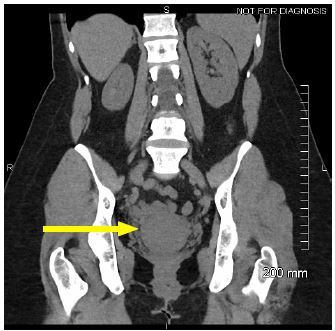
Computed tomography of the abdomen and pelvis in coronal view demonstrating a hypodense pelvic mass (arrow).

Medical records showed that a loop electrosurgical excision procedure was performed approximately 10 months prior. Labs were notable for white blood cell count of 8.14 × 10^9^/l (3.5–10.5), hemoglobin 13.8 grams per deciliter (13.5–17.5), hematocrit 42.2 % (38–50), and platelets 306 × 10^9^/l (150–450). A comprehensive metabolic panel and urinalysis were grossly normal. Urine human chorionic gonadotropin was negative. A computed tomography of the abdomen and pelvis was performed, which showed a hypodense mass in lower cervix (Image 1).

**Image 2:**
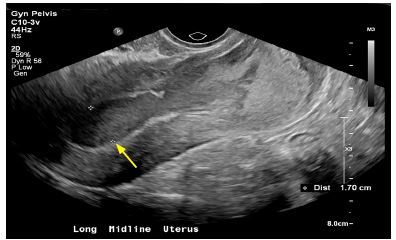
Formal pelvic ultrasound showing mixed echogenic contents contained within the uterus (arrow).

A formal pelvic ultrasound was performed and showed an enlarged uterus with mixed echogenic contents within the endometrial cavity likely representing hemorrhage of different ages and ongoing bleeding (Image 2). Gynecology was consulted and performed an incisional opening of the cervix and evacuated dark, mucoid blood and bright red blood. The patient was diagnosed with hematometra from scarred cervical os and was prescribed oral estrogen to prevent reclosure.

## DISCUSSION

Hematometra is a collection or retention of blood in the uterus most commonly due to an imperforate hymen or transverse vaginal septum.^1^ Acquired causes leading to cervical stenosis include radiation treatment, ablation, cervical conization, or malignancies.^2^ Diagnosis can be confirmed with pelvic exam and ultrasound. Treatment is usually done with surgical dilation. This diagnosis should be considered in females of child-bearing age with lower abdominal and pelvic pain.

**CPC-EM Capsule**

What do we already know about this clinical entity?

Hematometra is a rare but potential cause of pelvic pain in women, especially those who may have undergone cervical procedures leading to scarring.

What is the major impact of the image(s)?

Hematometra can be suspected when pelvic ultrasound shows mixed echogenic contents.

How might this improve emergency medicine practice?

In the patient presenting to the emergency department with pelvic pain, consider hematometra based on previous obstetrical and gynecological history.

## References

[1] Dolan MS, Hill C, Valea FA, Lobo R, Gershenson DM, Letz G, Valea F (2017). Benign gynecological lesions: vulva, vagina, cervix, uterus, oviduct, ovary, ultrasound imaging of pelvic structures. Comprehensive Gynecology.

[2] Hoffman BL, Schorge JO, Bradshaw KD (2016). Pelvic mass.

